# Application of structural allogenous bone graft in two-stage exchange arthroplasty for knee periprosthetic joint infection: a case control study

**DOI:** 10.1186/s12891-022-05228-6

**Published:** 2022-04-05

**Authors:** Chieh An Chuang, Sheng-Hsun Lee, Chih-Hsiang Chang, Chih-Chien Hu, Hsin-Nung Shih, Steve W. N. Ueng, Yuhan Chang

**Affiliations:** 1 Department of Orthopaedic Surgery, Chang Gung Memorial Hospital, 5, Fu-Hsin St., Kweishan, Taoyuan, Linko Taiwan; 2 Bone and Joint Research Center, Chang Gung Memorial Hospital, Linko Taoyuan, Taiwan; 3 College of Medicine, Chang Gung University, Taoyuan, Taiwan

**Keywords:** Revision knee arthroplasty, Periprosthetic joint infection, Bone defect, Structural allogenous bone graft

## Abstract

**Background:**

Knee prosthetic joint infection (PJI) is a common but devastating complication after knee arthroplasty. The revision surgeries for knee PJI may become more challenging when it is associated with large bone defects. The application of structural bone allograft in knee revision surgeries with large bone defects is not a new technique. However, there is a lack of literature reporting its efficacy in PJI cases. This study aimed to investigate the outcome of structural fresh frozen allogenous bone grafts in treating patients in knee PJI with large bone defects.

**Methods:**

We performed a retrospective cohort analysis of knee PJI cases treated with two-stage exchange arthroplasty at our institution from 2010 to 2016. 12 patients with structural allogenous bone graft reconstructions were identified as the study group. 24 patients without structural allograft reconstructions matched with the study group by age, gender, and Charlson comorbidity index were enrolled as the control group. The functional outcome of the study group was evaluated with the Knee Society Score (KSS). Treatment success was assessed according to the Delphi-based consensus definition. The infection relapse rate and implant survivorship were compared between groups.

**Results:**

Revision knees with structural allograft presented excellent improvement in the KSS (33.1 to 75.4). There was no significant difference between infection relapse-free survival rate and prosthesis survival rate in the two groups. The 8-year prosthesis survival rate was 90.9% in the study group and 91% in the control group (*p* = 0.913). The 8-year infection relapse-free survival rate was 80 and 83.3% in the study group and control group, respectively (*p* = 0.377).

**Conclusion:**

The structural fresh frozen allogenous bone graft provided an effective way for bone defect reconstruction in knee PJI with an accountable survival rate. Meanwhile, using structural allografts did not increase the relapse rate of infection.

## Background

Periprosthetic joint infection (PJI) is a devastating complication after total knee arthroplasty (TKA). The incidence of knee PJI is about 2% in primary TKA [1] and accounts for nearly 20% in revision cases [2]. Two-stage exchange arthroplasty, which provides an 85 to 95% of infection eradication rate with an improved functional score, has been recognized as the treatment of choice for PJI [3, 4].

The bone defect is a challenging problem in revision TKA, which may result from osteolysis, stress shielding, infection, or multiple revisions [5]. Managing this problem can vary widely depending on the defect size, patient condition, and surgeon experience [6, 7]. Allogenous bone graft is a common resolution for reconstructing bone defects in revision TKA that can fill bone defects and provide mechanical support [5–7]. The fresh-frozen allogenous bone graft can provide better biological and mechanical results than irradiated allogenous bone graft [8]. However, there are several concerns of using allogeneous bone grafts, including potential risks of infection, aseptic loosening, disease transmission, nonunion, and bone resorption [9, 10].

Hsieh et al. had conducted a study on using bone allografts to treat hip PJI with massive bone loss, showing positive results with no further infection [11]. However, limited literature is available on structural allogenous bone grafts in revision knee PJI with bone defects. Previous literature had reported the risk of infection by using allograft in revision knees [9]. Therefore, using structural allograft in knee revision PJI is still a controversial issue.

The purpose of our study was to examine the clinical and functional outcomes of knee PJIs by using structural allografts to reconstruct bone defects in a two-stage revision procedure.

## Methods

We retrospectively reviewed the joint arthroplasty databases to identify patients diagnosed with knee PJI and treated with two-stage exchange arthroplasty at our institution between January 2010 and December 2016. The study was approved by the Institutional Review Board of our institution according to the applicable laws and regulations.

PJI was defined by fulfilling one of the following criteria: (1) a sinus tract communicating with the prosthesis; (2) isolated pathogens in two or more samples obtained from the infected prosthetic joint; (3) Four out of six of the following findings: Presence of purulence in the affected joint; Elevated synovial white blood cell count; Elevated synovial polymorphonuclear percentage (PMN%); Elevated serum erythrocyte sedimentation rate (ESR) and serum C-reactive protein (CRP); Isolated pathogens in the sample obtained from the infected joint; Positive of histologic analysis from the periprosthetic tissue [12].

**Fig. 1:**
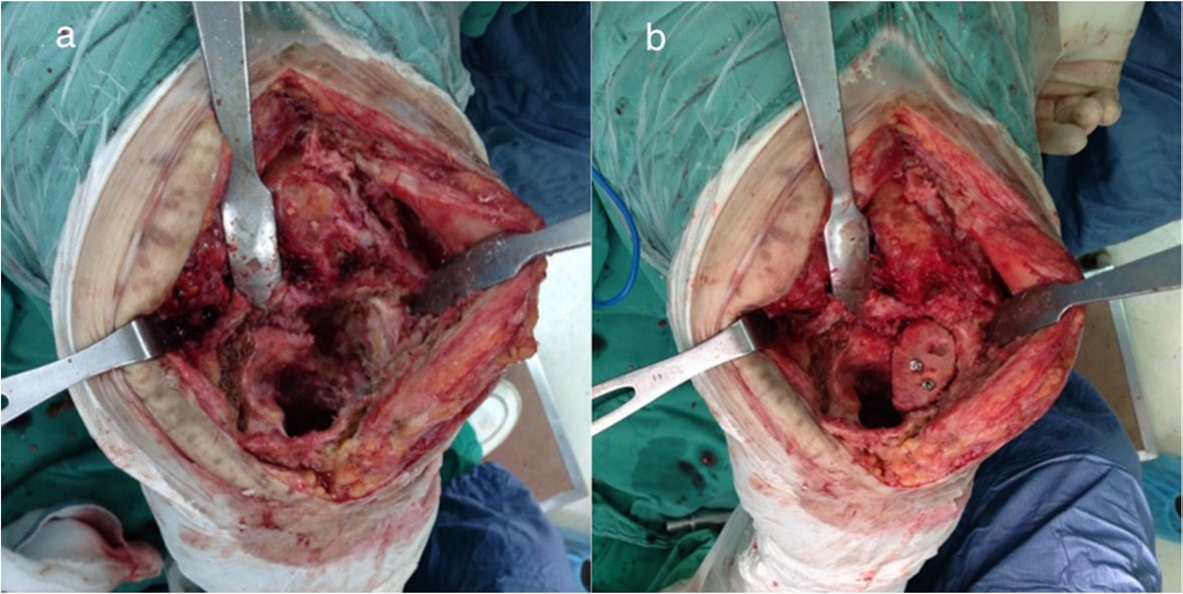
Intraoperative pictures from second-stage reconstruction of a 50 years old female diagnosed with PJI with bone defect showing **a**) AORI type IIB bone defect over left tibia **b**) defect reconstructed by structural allogenous bone graft with screws

All the enrolled patients were treated with two-stage exchange arthroplasty. In brief, resection arthroplasty included radical debridement, removal of the prosthesis, antibiotic-loaded bone cement implantation, and administration of systemic antimicrobial agents for controlling the joint infection in the interim period. The given antibiotics during the interim period differed depending on the patient’s culture results. We provided I.V. form empiric antibiotics initially after the 1st-stage operation. After that, we changed antibiotics specific to the culture once the laboratory reported the culture result. The I.V. form antibiotics would be given till a decreased and stable CRP level. We then shifted oral antibiotics for the patients if there were suitable and effective oral antibiotics for the culture result. The total period of oral and I.V. would be last for at least 6 weeks. On the other hand, if there were no suitable oral antibiotics with only certain I.V. antibiotics being effective to the culture result, the I.V. form antibiotics would be continued for at least 6 weeks during hospitalization. Delayed reimplantation of the prosthesis was performed after successful antimicrobial therapy, which was defined by the absence of signs of infection with ESR and CRP resumed to normal levels [13]. The bone defects were evaluated during the second stage revision surgery according to the Anderson Orthopedic Research Institute (AORI) bone defect classification [14]. The allografts were shaped into appropriate sizes then attached to the host bone defect site. We then filled the morselized allograft to residual space between the host bone and the structural allograft. After reconstruction of the bone defect, the prosthesis was implanted fully cemented. Figure 1 showed an example treatment case.

The indication we applied structural allograft were as follows:

1. Patients who sustained large bone defects (AORI IIA or IIB) with more than 20 mm in depth that could not be restored by metal augments. However, in controversial cases, we did try the metal augment first during our surgery if the bone defect was not as large in our initial evaluation. Once the bone defect was so large that it caused any instability or imbalance after the application of the metal augment trial, we would change to structural allograft reconstruction.

2. Patients sustained large and uncontained bone defect that was insufficient to support the metal cones (AORI III).

3. Patients presented with a contained but significant defect that was appropriate for the metal cone reconstruction but with financial difficulties. The cost of the metal cone is not covered by Taiwan Public Health Insurance.

### Control group

We randomly matched two patients without structural bone allograft reconstruction as the control group (non-allograft group) to each patient from the study group (allograft group) based on age, gender, and Charlson comorbidity index (CCI). The paired patients were from the knee joint PJI data in our institute.

### Bone graft

The source of allografts was from the bone bank in our institute. We used deep freezing to sterilize the allograft bone and eliminate antigen-antibody reactions between the allograft and the hosts. The protocol of allograft bone-retrieving was the same as described by Wu et al. in our institute [15].

### Outcome assessment

The function of the involved knees was assessed before operation and at OPD follow-up according to Knee Society Score (KSS) [16]. Treatment success was assessed according to the Delphi-based consensus definition, including (1) infection eradication, characterized by a healed wound without fistula, drainage or pain, and no infection recurrence caused by the same organism strain; (2) no post-operative infection after reimplantation surgery; and (3) no occurrence of PJI-related mortality with an at least 2-year follow-up.

The radiographs were examined regularly for evidence of allograft resorption, migration, or loosening of the allograft-prosthesis composite. The incorporation of the allograft was determined by the appearance of trabecular remodeling within the grafted area and the disappearance of the gap between the host and the allograft.

The Log-rank test was performed for Kaplan-Meier survival curve analysis. Statistical analysis was conducted by an independent statistician blinded to surgical outcomes. Statistical analysis was performed with SPSS software version 23.0.

## Results

Between January 2010 and December 2016, we identified 157 consecutive patients with knee PJI treated by a 2-stage exchange arthroplasty protocol at our institute. Patients who did not meet the minimal 2-year-follow up were excluded. At last, 22 patients could not meet the 2-year follow-up, and 135 patients were enrolled. Among the lost 22 patients, two patients had structural allografts used in their knee reconstructions. Both of them passed away owing to internal medicine comorbidities within 2 years after the knee revision surgeries. However, they presented well-functioned status with no infection during their final follow-up at the orthopedic outpatient department. Among the enrolled 135 patients, 12 patients with massive bone loss treated with fresh frozen structural bone allograft during 2nd stage reconstruction were enrolled as our study group. There were ten right knees and two left knees. Their mean age at revision was 68.1 years old (from 45.7 to 81.1), and the mean follow-up time was 62.5 months (from 25.7 to 95.0). Details of the 12 allograft-treated patients were shown in Table 1 and their infection diagnosis information was shown in Table 2. The mean age of the 24 patients in the control group was 68.1 years old (from 32.9 to 82.7), the mean follow-up time was 58.4 months (from 24.7 to 129.4). Details of the control group were shown in Table 3 and their infection diagnosis information was shown in Table 4.

**Table 1:** The characteristics of patients in the allograft group (Study group)

No.	Gen	Age	Status before Infection	Comorbidities	CCI	Bone defect (AORI)	Allograft Applied Site	Prosthesis	Abx in BCS	1st stage culture	Abx in interim period
1	F	64.5	Revision TKA	DM	3	F: III T: I	Femur	United, U2 PSA	Vancomycin+ceftazidime	*Staphylococcus epidermidis*	Cotrimoxazole
2	M	67.3	Revision TKA	DM, HTN, CKD	5	F: IIB	Femur	United, U2 PSA	Vancomycin+ceftazidime	*Staphylococcus aureus* (OSSA)	Clindamycin
3	F	79.0	Revision TKA	DM, HTN, CAD	5	F: IIB	Femur	United, U2 PSA	Vancomycin+ceftazidime	*Staphylococcus aureus* (OSSA)	Dicloxacillin
4	M	65.9	Primary TKA	DM, HTN	3	F: III	Femur	Zimmer, LCCK	Vancomycin+ceftazidime	*Staphylococcus aureus* (OSSA)	Dicloxacillin
5	F	79.0	Primary TKA	HTN	3	F: III	Femur	United, U2 PSA	Vancomycin+ceftazidime	*Enterococcus faecalis*	Ampicillin
6	M	78.2	Revision TKA	HTN	3	F: I T: III	Tibia	Zimmer, LCCK	Vancomycin+ceftazidime	*Enterococcus faecalis*	Sodium fusidate
7	F	50.4	Revision TKA	SLE	2	T: IIB	Tibia	Zimmer, RHK	Vancomycin+ceftazidime	*Pseudomonas aeruginosa*	Ciprofloxacin
8	M	63.5	Primary TKA	DM, RA	4	F: III	Femur	Zimmer, LCCK	Vancomycin+ceftazidime	*Serratia marcescens*	Ciprofloxacin
9	F	71.8	Primary TKA	DM	4	F: IIB T: I	Femur	United, U2 PSA	Vancomycin+ceftazidime	*Mycobacterium tuberculosis*	Rifampicin+Isoniazid
10	F	81.1	Primary TKA	HTN, CVA	5	F: I T: IIB	Tibia	United, U2 PSA	Vancomycin+ceftazidime	*Escherichia coli*	Ciprofloxacin
11	F	45.7	Primary TKA	DM, HB	2	F: III	Femur	United, tumor prosthesis	Vancomycin+ceftazidime	*Staphylococcus aureus*	Dicloxacillin
12	F	70.2	Primary TKA	HTN	3	T: III	Tibia	Zimmer, LCCK	Vancomycin+ceftazidime	Coagulase(−) staphylococcus	Dicloxacillin, cotrimoxazole

*Gen* Gender, *CCI* Charlson comorbidity index, *TKA* Total knee arthroplasty, *Abx* Antibiotics, *BCS* Bone cement spacer, *F* Femur, *T* Tibia, *HTN* Hypertension, *DM* Diabetes mellitus, *CKD* Chronic kidney disease, *CAD* Coronary artery disease, *HB* Hepatitis B, *RA* Rheumatic arthritis, *SLE* Systemic Lupus Erythematosus, *OSSA* Oxacillin- susceptible *Staphylococcus aureus*, *Zimmer* Zimmer Biomed Institute, USA, *United* United Orthopedics corporation, Taiwan

**Table 2:** The PJI diagnosis information of the study group

No.	Sinus tract	Sets of positive synovial culture	Serum WBC (/ul)	Serum CRP (mg/L)	Serum ESR (mm/hr)	Synovial WBC (/ul)	Synovial PMN (%)
1	positive	2	9600	28.94	56	6723	92
2	positive	2	16,300	161.22	134	8625	97
3	negative	1	7300	20.6	44	8986	94
4	positive	2	13,200	67.32	53	7348	88
5	positive	2	7200	51.45	63	6573	90
6	negative	2	5800	58.8	89	18,500	93
7	positive	1	6900	20.8	35	7726	87
8	negative	2	7800	14.68	113	2560	92
9	positive	1	8400	19.55	38	12,454	87
10	negative	2	6600	46.6	70	14,125	92
11	Positive	2	9300	140.6	117	9415	93
12	Negative	2	10,800	39.68	33	12,020	95

*WBC* White blood cell, *CRP* C-reactive protein, *ESR* Erythrocyte sedimentation rate, *PMN* Polymorphonuclear leukocytes

**Table 3:** The characteristics of patients in the non-allograft group (Control group)

No.	Gen	Age	Status before Infection	Comorbidities	CCI	Bone defect (AORI)	Bone defect management	Prosthesis	Abx in BCS	1st stage culture	Abx in interim period
1	F	62.7	Primary TKA	Cecal cancer	2	Nil	Nil	Zimmer, LCCK	Vancomycin+piperacillin	Ps.stutzeri	Ciprofloxacin
2	F	62.3	Primary TKA	HTN, DM	3	F: IIA	F: Metal augment	United, PSA	Vancomycin+ceftazidime	Staph.aureus (ORSA)	Daptomycin, Ciprofloxacin
3	M	65.6	Revision TKA	HTN, DM, CKD, HC	5	T: I	T: Bone cement	United, PSA	Vancomycin+ceftazidime	Nil	Vancomycin
4	M	68.2	Primary TKA	HTN, DM, PU, CVA,	5	Nil	Nil	Zimmer, LCCK	Vancomycin+ceftazidime	*Serratia marcescens*	Ciprofloxacin
5	F	77.6	Revision TKA	HTN, DM, CKD	6	F: III T: III	Custom-made prosthesis	Custom-made prosthesis	Vancomycin+ceftazidime	Nil	Vancomycin
6	F	80.1	Primary TKA	HTN, DM	5	F: IIA	F: Metal augment	United, PSA	Vancomycin+ceftazidime	Nil	Dicloxacillin
7	M	63.2	Revision TKA	HTN	2	F: IIB	F: Metal augment	Zimmer, RHK	Vancomycin+ceftazidime	Ps.aeruginosa	Fortum
8	M	65.3	Primary TKA	HTN, HB	3	Nil	Nil	United, PSA	Vancomycin+ceftazidime	Staph.epidermidis (ORSE)	Vancomycin
9	F	79.1	Revision TKA	nil	3	F: IIA T: IIA	F: Metal augment T: Metal augment	United, PSA	Vancomycin+ceftazidime	Staph.aureus (OSSA)	Dicloxacillin
10	F	76.0	Primary TKA	HTN, DM	4	F: I T: I	F: Bone cement T: Tibia cement	Zimmer, RHK	Vancomycin+ceftazidime	Staph.aureus (ORSA)	Cotrimoxazole
11	M	77.2	Primary TKA	HTN	3	F: IIA	F: Metal augment	United, PSA	Vancomycin+ceftazidime	Staph.aureus (OSSA) Ecoli	Dicloxacillin
12	M	80.3	Revision TKA	Asthma	4	F: I T: I	F: Bone cement T: Bone cement	United, PSA	Vancomycin+ceftazidime	B-strepto. Gr.A Gm(−) bacilli-glucose nonfermentin	Ampicillin
13	F	59.3	Revision TKA	nil	1	F: I	F: Bone cement	United, PSA	Vancomycin+ceftazidime	Staph.epidermidis (ORSE)	Rifampicin Fusidin
14	F	50.8	s/p ITL nail	DM	2	Nil	Nil	United, PSA	Vancomycin+ceftazidime	Staph. epidermidis (ORSE)	Rifampicin Fusidin
15	M	62.1	Revision TKA	HTN, HB	3	F: IIA T: III	F: Metal augment T: TM cones	United, PSA	Vancomycin+ceftazidime	Staph.aureus (OSSA)	Dicloxacillin
16	M	60.4	Primary TKA	RA	3	F: IIB T: IIA	F: Metal augment T: Metal augment	Zimmer, RHK	Vancomycin+ceftazidime	Staph. epidermidis (ORSE)	Clindamycin
17	F	74.9	Primary TKA	HTN, DM	4	Nil	Nil	United, PSA	Vancomycin+ceftazidime	Nil	Teicoplanin, ertapenem
18	F	74.6	Revision TKA	HTN, PU, HB	5	F: III T: IIA	Custom-made prosthesis	Custom-made prosthesis	Daptomycin+teinem	*Parvimonas micra*	Metronidazole
19	F	82.7	Primary TKA	HT, DM, CVA	6	F: IIA	F: Metal augment	Zimmer, LCCK	Vancomycin+ceftazidime	Staph.aureus (OSSA)	Rifampicin
20	F	79.8	Primary TKA	HT, DM Dementia, CAD	6	Nil	Nil	United, PSA	Vancomycin+ceftazidime	Kleb pneumoniae	Ampicillin
21	F	56.8	Revision TKA	HB	2	F: III T: IIB	Custom-made prosthesis	Custom-made prosthesis	Vancomycin+ceftazidime	Ps.aeruginosa	Ciprofloxacin
22	F	32.9	Revision TKA	Bone tumor s/p	2	F: III T: III	Custom-Made prosthesis	Custom-Made prosthesis	Vancomycin+ceftazidime	Staph.aureus (ORSA)	Teicoplanin
23	F	69.9	Primary TKA	HTN	2	F: IIB T: IIA	F: Metal augment T: Metal augment	United, PSA	Streptomycin	mycobacterium tuberculosis complex	Rifinah
24	F	71.9	Revision TKA	HTN	3	F: III T: III	Custom-Made prosthesis	Custom-Made prosthesis	Vancomycin+ceftazidime	Staph.aureus (OSSA)	Dicloxacillin

*Gen* Gender, *CCI* Charlson comorbidity index, *TKA* Total knee arthroplasty, *Abx* antibiotics, *BCS* Bone cement spacer, *s/p* status post, *ITL* Interlocking nail, *F* Femur, *T* Tibia, *HTN* Hypertension, *DM* Diabetes mellitus, *HB* Hepatitis B, *HC* Hepatitis C, *CKD* Chronic kidney disease, *CVA* Cerebrovascular accident, *CAD*: coronary artery disease, *PU* Peptic ulcer, *Gm(−)* Gram negative, *Ps* Pseudomonus; *Staph* Staphylococcus, *OSSA* Oxacillin- susceptible *Staphylococcus aureus*, *ORSA* Oxacillin-resistant *Staphylococcus aureus*, *ORSE* Oxacillin-resistant *Staphylococcus epidermidis*, *Zimmer* Zimmer Biomed Institute, USA; *United* United Orthopedics corporation, Taiwan

**Table 4:** The PJI diagnosis information of the control group

No.	Sinus tract	Sets of positive synovial culture	Serum WBC (/ul)	Serum CRP (mg/L)	Serum ESR (mm/hr)	Synovial WBC (/ul)	Synovial PMN (%)
1	negative	1	13,100	236	55	9367	92
2	negative	2	22,900	205.8	72	25,372	95
3	positive	nil	7800	149.9	54	80,163	91
4	negative	2	8200	82.2	45	15,683	92
5	negative	nil	8400	86	119	17,943	94
6	positive	nil	9400	17.43	51	9764	89
7	negative	2	8300	56.52	59	29,420	93
8	negative	2	10,100	31.1	63	30,400	93
9	negative	2	14,600	219.2	91	32,480	86
10	negative	2	6900	60.5	74	10,360	93
11	negative	2	9800	209	36	24,890	92
12	negative	2	16,400	368	87	15,125	96
13	negative	2	8700	56	77	8942	91
14	positive	2	12,300	193.8	97	21,040	94
15	negative	2	7600	96.5	74	17,764	92
16	negative	2	8900	64.35	54	23,250	89
17	negative	nil	14,200	74.92	48	12,500	99
18	negative	2	10,200	54.92	43	9526	93
19	positive	2	10,400	46.58	62	31,260	93
20	positive	1	7000	201.8	111	Nil	Nil
21	negative	2	6300	27.1	38	12,326	94
22	positive	2	8700	39.1	44	Nil	Nil
23	negative	2	9300	81.7	52	6380	42
24	negative	2	9900	85.13	56	19,860	89

*WBC* White blood cell, *CRP* C-reactive protein, *ESR* Erythrocyte sedimentation rate, *PMN* Polymorphonuclear leukocytes

The micro-organism isolated from the first stage of resection arthroplasty, the impregnated antibiotics in cement spacer and the provided antibiotics in the interim period were shown in Table 1 and Table 3. Generally, gram-positive bacteria account for the majority of the infection source.

**Fig. 2:**
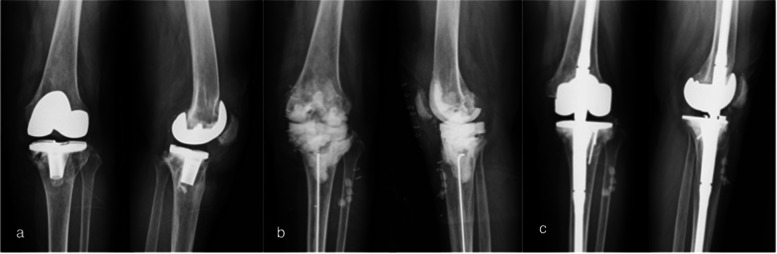
Radiographs of a 50-year-old female showing **a**) septic status of a left total knee arthroplasty **b**) 1 months after the first-stage operation with large tibia defect and reconstructed with antibiotics cement prosthesis and **c**) 2 years after revision with reconstruction of the tibia plateau defect restored by a femoral head allograft in structural type

The mean KSS improved from 33.1 (16–45) pre-operatively to 75.4 (49–87) postoperatively in the study group. The treatment example was showed in Fig. 2. The radiographs showed no nonunion or fracture in the allografts, but one graft resorption was noted during the follow-up. There was one patient who sustained re-infection, and one patient underwent implant revision owing to the bone graft resorption mentioned above during the follow-up. The details of the two patients are shown below.

**Fig. 3:**
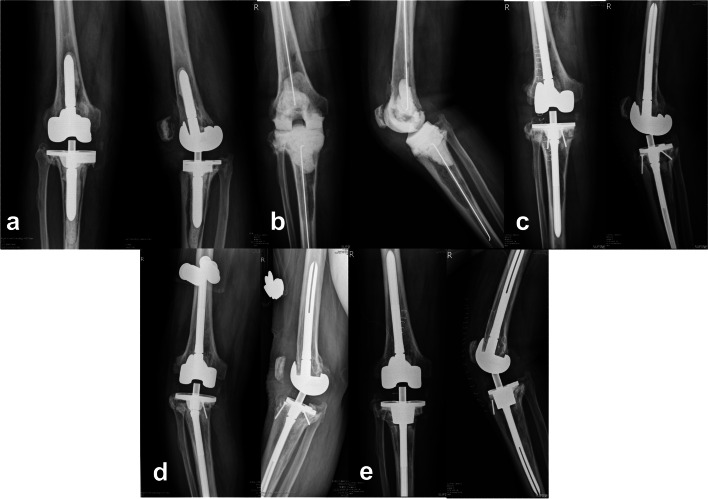
Radiographs of a 78-year-old male showing **a**) septic status of a left total knee arthroplasty **b**) status after 1st-stage ALBCS implantation and **c**) status after 2nd stage revision with reconstruction of structural allograft **d**) tibia component breakage with allograft resorption 32 months after revision **e**) status after revision surgery with TM cone reconstruction

One 50-year-old female patient reconstructed with tumor prosthesis at the 2nd stages surgery suffered from recurrent right knee infection at 74 months after 2nd stage reconstruction. Associated findings included urinary tract infection and left foot cellulitis. She was admitted to the infection ward for sepsis control first, and the operation with debridement and irrigation was performed later by us. The intra-operative culture was negative. After debridement surgery, we provided intravenous antibiotics (vancomycin and ceftriaxone) then oral antibiotics (dicloxacillin and clindamycin) for further infection control. Decreased CRP levels with improved clinical conditions were noted afterward. Another 78-years-old male sustained right knee soreness at 32 months after 2nd stage reconstruction. Tibia component breakage with bone graft resorption were noted from the radiographic follow-up. Revision surgery with TM cone implantation over the tibia side was performed with no infection sign noted intra-operatively. The radiographic image was presented as Fig. 3.

**Fig. 4:**
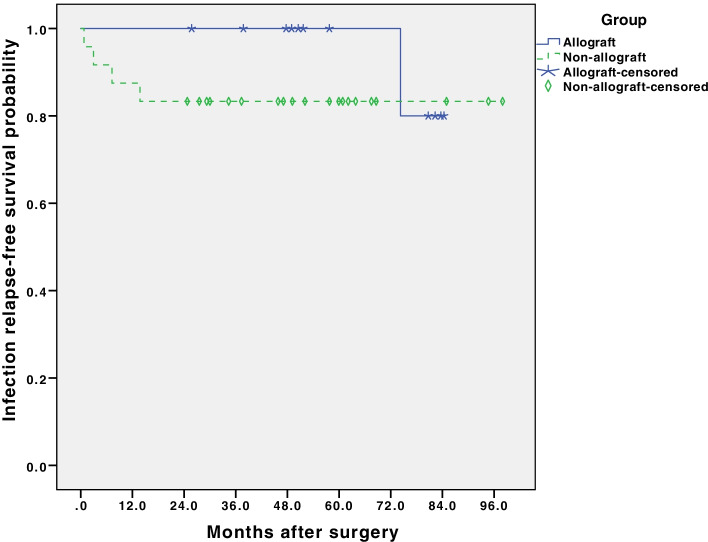
Kaplan-Meier survival curves show no significant difference in 8-year infection relapse-free survival rate between structural allograft group and non-structural allograft group in knee PJI revisions. (*p* = 0.377)

**Fig. 5:**
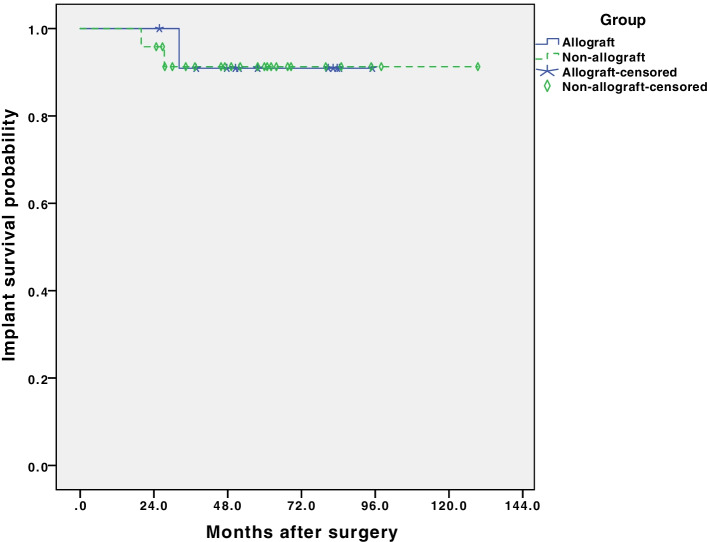
Kaplan-Meier survival curves show no significant difference in 8-year implant survival rate between structural allograft group and non-structural allograft group in knee PJI revisions. (*p* = 0.913)

The 8-year infection-free survival rate was 80% in the study group and 83.3% in the control group. There was no significant difference in 8-year infection-free survival between patients with or without allografts (*p* = 0.377, Fig. 4). For prosthesis survivorship, the 8-year prosthesis-retention survival rate was 90.9% in the study group and 91.0% in the control group (Fig. 5). There was no significant difference (*p* = 0.913) between these two groups.

## Discussion

Knee PJI accompanied with bone loss is a challenging problem in knee joint revision. Several studies have reported the outcomes of revision knees with bone defects reconstructed by bone allografts. However, limited literature focuses on the same issue in knee PJI cases. Using allogenous bone graft for bone defect reconstruction remains a controversial issue, especially for knee PJI revision. In this current study, we compared the outcomes of knee PJI with bone defect reconstruction by structural allografts to outcomes of general cases in knee PJI without using structural allografts. There was no significant difference in the relapse rate of infection and implant survival rate between the groups. We believe using structural allogenous bone graft in the second stage of knee PJI reconstruction can be safe and feasible.

### Two-stage treatment for knee PJI

PJI is one of the most common complications that leads to the revision of TKA [2], and two-stage total knee revision is considered as the proper treatment for PJI with a success rate of around 85–90% [3, 17, 18]. Bongers et al. [19] reported a 20% re-infection rate at 5 years in 113 PJI knees with 2-stage revision. We performed a 2-stage revision protocol for all knee PJI patients from our database including knees with bone defects. In our results, both groups presented with around 90% of 8-year prosthesis survival rate and approximately 80% of 8-year infection relapse-free survival rate. The outcome of 2-stage treatment for knee PJI was in line with previous studies.

### Bone defect management

Bone defects reconstruction is challenging in knee revision arthroplasty [20]. When it comes to large bone defects, metal augment is feasible in managing AORI type II bone defects, and structural allograft is the treatment option for AORI type IIB to type III [5, 7]. Hockman et al. [21] compared the efficacy of using metal augment to allograft for bone loss treatments in knee revision. The result revealed a 59% failure rate of using metal augment alone, and 48% of the knees required additional structural allograft. It was believed that metal augment reduced the contact surface between the host bone and the implant, making it relatively more unstable. In addition, augment could not manage bone defects over 20 mm depth, and there were risks of fretting and erosion, as well as limitations on bone stock restoration [6, 22]. Richards et al. [23] also declared that patients treated with allograft demonstrated better clinical outcomes and lower complication rates than those treated with metal augment.

Metal cones were another treatment choice for knee revision with large bone defects as AORI type III. A systematic review had reported a lower loosening rate of the porous metal cones than structural allograft in revision knees [24]. However, the re-infection rate revealed no significant difference between these two methods. In our experiences, it was necessary to remove additional host bones when applying metal cones in particular cases. Besides, metal cones have a limited choice in sizes and are inapplicable to knees with small bone sizes. Furthermore, metal cones may cause an additional financial burden to patients comparing to allograft, which is more cost-effective. All these factors need to be considered when managing large bone defects indicated for both structural allograft and cones systems.

### Structural allogenous bone graft

Structural allograft is known for its capability of incorporating the host bone and stress protection. Literature had reported a prosthesis survival rate between 76 and 93% at a 5-year follow-up, showing the capability of structural allograft in treating knee revision cases with bone loss [9, 10, 25]. For mid-term to long-term survivorship, Chun et al. [26] reported the results of 27 patients undergoing revision TKA with severe bone defect using a fresh-frozen femoral head allograft with a minimum of 8-year follow up. They demonstrated an improved Hospital for Special Surgery knee score from 46 to 83, and 26 out of 27 patients presented no complications. Engh et al. reported a 91% survivorship at 10 years for femoral head allograft in tibial defects in 46 patients receiving revision TKA [27]. Clatworthy et al. presented a prosthesis survival rate of 92% at 5 years and 72% at 10 years in 52 patients with uncontained bone defect constructed with structural allograft in knee revisions [28]. In this current study, cases enrolled in the study group were all knee PJI cases with bone defects. We demonstrated a 90.9% 8-year implant survival rate in structural allograft reconstruction knees, indicating a similar outcome compared with the previous literatus though our cases were all PJI revision knees.

Despite the reliable results of structural allograft in revision knees, some complications were still with concerns. The potential risk of disease transmission remains an unresolved problem of using bone allografts [29]. Several studies had reported a higher infection rate of using allograft bone, which led to revision failure. Franke et al. reported a study in which 30 patients were treated with allografts for revision TKA, and the infection rate was 10% [10]. Bauman et al. reviewed structural allograft for TKA reconstruction with an infection rate of 7.1% [9]. Backstein et al. used structural allograft in revision TKA and reported a re-operation rate of 4.9% for secondary infection [30]. However, Wang et al. conducted a case series study with revision knee reconstructed with femoral head allograft showed no recurrent infection among PJI cases [31]. In this current study, one patient in the study group sustained recurrent infection, while the other 11 patients presented free of infection relapse. The results showed an infection rate of 8.3%, and a 100% 5-year infection-free survival rate and an 80% 8-year infection-free survival rate. Though the infection rate was similar to previous articles, it was acceptable owing to cases in this study were all PJI cases. We supposed the application of structural allograft to knee PJI cases did not increase the infection rate compared to general cases. The outcome can be attributed to the following reasons. First, the bone bank in our institute is under strict regulations which are supervised by the government, and all of the bone grafts were harvested by experienced surgeons [15]. Wu et al. had reported the result of using bone allografts from our bone bank for surgery with a relatively low infection rate of 1.2% [15]. Second, all allografts used in this study were femoral heads harvested from the patients undergoing total hip arthroplasty in our institute. Based on our previous study, administering prophylaxis antibiotics before surgery resulted in their presence in fresh-frozen femoral heads, which exhibited inhibitory effects against bacteria in vitro after 2 weeks of deep-frozen storage [32].

For other complications, Backstein et al. reported three cases of resorption and one case of nonunion in 58 patients [30]. Franke et al. reported one case of nonunion to the graft and host bone in 30 cases of revision knee using allograft bone [10]. Bauman et al. reported two cases of nonunion and three cases of post-operative fracture in 79 cases of revision knee treated by structural bone allograft [9]. In our study, no nonunion or fracture was reported during our radiographic follow-up concerning structural allograft. However, one patient (8.3%) suffered from tibial component breakage two and half years after the reconstruction surgery, and the structural allograft applied to the tibia bone defect showed resorption by x-ray follow-up.

By comparing our study group results to the control group (PJI cases without allografts) and previous articles on revision knees managed with structural allograft, the results revealed the capability of allograft to resolve bone defects in knee PJI cases. The outcome showed no additional infection relapse rate and a satisfying prosthesis survival rate. This study validated the efficacy of using allograft in solving PJI with bone loss in the two-stage revision procedure.

### Limitations

The study has several limitations. First, this was a retrospective study with a relatively small sample size. Second, the size of the control group’s bone defects was inconsistent with that of the study group. Some of the cases in the control group might be simple revision cases that presented with minimal bone defects. Generally, the bone defect would be more extensive in the study group. The infection rate in complex revisions knees, which require structural bone reconstruction, might be higher than simple revision or small bone defect cases. However, we presented a different point of view according to our results. Third, we did not compare the functional outcome between the study and control groups, which is also essential for comparison. Nevertheless, the overall KSS was good in our study group after the revisions. At last, the implants were not matched between cases in the study group and control group. The different implants might affect the survival rate and complication rate.

## Conclusion

The reconstruction of bone defects in knee PJI with structural allogenous bone graft does not increase the relapse rate of infection and provides a good prosthesis survival rate. The use of structural fresh frozen allogenous bone graft for managing bone defects in the second stage of knee PJI reconstruction is a promising and safe method.

## Data Availability

The datasets generated during or analysed during the current study are available from the corresponding author on reasonable request.

## Abbreviations

PJI: Prosthetic joint infection
PMN%: Polymorphonuclear percentage
ESR: Erythrocyte sedimentation rate
CRP: C-reactive protein
AORI: Anderson Orthopedic Research Institute
CCI: Charlson comorbidity index
KSS: Knee Society Score
